# Design of Adaptive Fractional-Order Fixed-Time Sliding Mode Control for Robotic Manipulators

**DOI:** 10.3390/e24121838

**Published:** 2022-12-16

**Authors:** Saim Ahmed, Ahmad Taher Azar, Mohamed Tounsi

**Affiliations:** 1 College of Computer and Information Sciences, Prince Sultan University, Riyadh 11586, Saudi Arabia; 2 Automated Systems and Soft Computing Lab (ASSCL), Prince Sultan University, Riyadh 11586, Saudi Arabia; 3 Faculty of Computers and Artificial Intelligence, Benha University, Benha 13518, Egypt

**Keywords:** robotic manipulators, adaptive fixed-time control, fractional-order sliding mode control, unknown dynamics

## Abstract

In this investigation, the adaptive fractional-order non-singular fixed-time terminal sliding mode (AFoFxNTSM) control for the uncertain dynamics of robotic manipulators with external disturbances is introduced. The idea of fractional-order non-singular fixed-time terminal sliding mode (FoFxNTSM) control is presented as the initial step. This approach, which combines the benefits of a fractional-order parameter with the advantages of NTSM, gives rapid fixed-time convergence, non-singularity, and chatter-free control inputs. After that, an adaptive control strategy is merged with the FoFxNTSM, and the resulting model is given the label AFoFxNTSM. This is done in order to account for the unknown dynamics of the system, which are caused by uncertainties and bounded external disturbances. The Lyapunov analysis reveals how stable the closed-loop system is over a fixed time. The pertinent simulation results are offered here for the purposes of evaluating and illustrating the performance of the suggested scheme applied on a PUMA 560 robot.

## 1. Introduction

The latest advancements in the domain of control systems are having a significant impact on the field of mechatronics and robotic system design and development. The topic of controlling a robotic manipulator is investigated in the field of control theory. Specifically, it is a highly non-linear system that also possesses a high degree of mechanical instability. Due to this, the system in question needs to be able to maintain a high level of stability, while still having the capacity to monitor accurately its course in the face of external disturbance and uncertainty [1]. Despite the fact that a large variety of viable solutions have been proposed for uncertain robotic systems that are subject to external disturbances, it is impossible to avoid the uncertain parameters when operating under real-world conditions. Due to this, it is difficult for a system to be precisely regulated if the controller is impacted in any way by the disturbance. As a direct result of this, there is a growing interest in the creation of robust control systems, which have been the subject of substantial research and are currently being deployed in a wide variety of industries [2]. Moreover, a robust adaptive control mechanism is built to compensate for the unknown uncertainties and disturbances so that the system continues to function effectively. The advantage of the approach behind robust adaptive control is that the control system itself needs to be robust in order to guarantee the attainment of the necessary level of both performance and stability.

Sliding mode control, commonly known as SMC, is a type of control strategy that is both non-linear and robust [3]. It can effectively deal with non-linear systems that are uncertain, have confined disturbances, and have a low sensitivity to changes in the system’s parameters. Terminal SMC (TSMC) was introduced in [4] with the objective of achieving robust finite-time stability. TSMC offers accurate tracking and increased precision. However, delayed convergence and singularity are problematic. As a result, SMC approaches were created as solutions to these issues in order to achieve rapid convergence with fast terminal SMC (FTSMC) and eliminate singularities with non-singular terminal SMC (NTSMC) [5,6]. Moreover, the initial values of the non-linear system have a significant impact on the amount of time required for the finite-time system to converge, and this amount of time always increases as the initial values of the non-linear system increase. Fixed-time stability is, therefore, an option that can be utilized to precisely compute the time of convergence irrespective of the initial conditions [7,8].

The theory of fractional-order (Fo) calculus, which has been around for the past three centuries and deals with derivatives and integrals of non-integer order [9,10,11,12,13], was recently rediscovered by scientists and engineers and is being utilized in various domains such as material sciences [14], bioengineering [15], finance [16], and electronic circuits [17,18], including the field of control theory [19,20,21,22,23,24]. The numerous control techniques such as proportional–integral–derivative (PID) control, the SMC method, and various fuzzy and neural network schemes have all implemented their respective control techniques using a Fo controller [25,26,27,28,29]. Dadras [30] is credited with being the first author to present the ideas of Fo in combination with finite-time TSMC. Moreover, the adaptive scheme with fractional-order non-singular fast TSMC (FOTSMC) was introduced with the intention of controlling the robotic manipulator. This was done so as to address the issue of dealing with unknown dynamics [31]. Recently, several Fo fixed-time SMC schemes have been developed for applications such as micro-gyroscopes [32], chaotic systems [33], unmanned surface vessesl [34], nonholonomic mobile robots [35], and multimachine power systems [36].

Control engineering applications are increasingly gravitating toward the use of adaptive control, which is a well-known control technology that is gaining popularity [37,38]. It demonstrates an unusual capacity for adaptation in the face of system uncertainty and external disturbances, and it helps improve the tracking performance of closed-loop systems [39,40]. A robust adaptive strategy based on a class of high-order SMC was devised for a fractional chaotic system in the presence of non-linearity [41]. Several adaptive finite-time FoSMC techniques have been suggested for use with the robotic manipulator, which also takes into account the presence of uncertainties and disturbances. In the study in [25], a robust adaptive finite-time FoFTSM was built for the robotic system. Within this model, unknown dynamics were estimated by employing an adaptive controller. It was suggested to estimate the unknowable dynamics of the non-linear robot using an output feedback adaptive super-twisting finite-time FoSMC [31]. Moreover, a fixed-time disturbance observer-based adaptive FoNFTSM has been designed for indeterminate manipulators under unknown disturbances [42].

It is fascinating to note that each of the aforementioned papers concentrated their attention largely on the adaptive scheme for the estimate of the upper bounds of uncertain dynamics by applying finite-time FoNTSM control. It is generally agreed that the most significant benefit of using fixed-time non-singular TSMC (FxNTSM) control is that it eliminates the risk of singularity, possesses high robustness in the face of both internal and external disturbances, and ensures that convergence time is independent of the initial values. This research has shown that very few works provide adaptive FxNTSM control, and that no research whatsoever has been conducted on adaptive FoFxTSMC. Within the scope of this study, the fixed-time convergence of robotic manipulator systems that are vulnerable to external disturbances is explored. Specifically, the research focuses on the effects of the unknown dynamics of the systems. Considering all of this, the adaptive fractional-order fixed-time non-singular terminal SMC is designed, which is also known as AFoFxNTSM, for uncertain robotic manipulators that are influenced by external disturbances. The most important contributions given by this work are organized into the following points:

1. Based on the characteristics of fractional-order fixed-time non-singular terminal SMC, a sliding surface with good tracking performance, reduced control input chattering, and rapid convergence is designed.
2. The fractional-order control is applied in an attempt to improve the performance of the closed system.
3. It is proposed to use adaptive control with FoFxNTSM, so that the unknown dynamics are compensated for in order to produce the robust and sustainable performance for the PUMA 560 robotic manipulator.
4. The Lyapunov theory is utilized in order to carry out an investigation into the system’s fixed-time stability.

The remaining parts of this work are organized as follows: The preliminaries are presented in Section 2. The modeling of the system, the control design, and its stability are explained in Section 3. The adaptive control approach and its stability are presented in Section 4. The numerical simulations to validate the performance of the proposed method are presented in Section 5. Section 6 is devoted to discussing the simulation findings. Section 7 delivers the conclusion of the paper.

## 2. Preliminaries

**Definition** **1.** *For fractional calculus, the Riemann–Liouville (RL) definition is often employed [43]. Consequently, the Fo integral and derivative are given as follows. The following equation gives the RL fractional integral, as well as the derivative of the $\alpha_{th}-order$ function f(t) in relation to t and a, provided by*

(1) $${}_{a}I_{t}^{\alpha }f\left(t\right)=\frac{1}{\Gamma (\alpha )}\int_{a}^{t}\frac{f(\tau )}{{\left(t-\tau \right)}^{1-\alpha }}d\tau$$

(2) $${}_{a}D_{t}^{\alpha }f\left(t\right)=\frac{d^{\alpha }f\left(t\right)}{dt^{\alpha }}=\frac{1}{\Gamma (1-\alpha )}\frac{d}{dt}\int_{a}^{t}\frac{f(\tau )}{{\left(t-\tau \right)}^{\alpha }}d\tau$$

*where n−1<α<n, m∈N and Γ(·) is the Gamma function, described by Euler as*

$$\Gamma \left(\alpha \right)=\int_{0}^{\infty }e^{-t}t^{\alpha -1}dt$$

*whereas D and I represent, respectively, the fractional integral and the derivative of the function.*

**Lemma** **1.** *Consider the following non-linear system [44]*

(3) $$\dot{x}\left(t\right)=f\left(t,x\right),\;\;\;x\left(0\right)=x_{0}$$

*where f(t,x) is a continuous non-linear function. For fixed-time stability with fast time convergence, the Lyapunov function V(x) satisfies that*

*a.* 
V(x)=0⇔x=0

*b.* 
$\dot{V}\left(x\right)\leq -\xi_{1}V^{\eta_{1}}\left(x\right)-\xi_{2}V{\left(x\right)}^{\eta_{2}}$

*where $\xi_{1},\;\xi_{2}>0,\;0<\eta_{1}<1\;\;\;$ and $\;\;\eta_{2}>1$. Then, the system is fixed-time stable and the convergence time can be computed as*

(4) $$T\leq \frac{1}{\xi_{1}\left(1-\eta_{1}\right)}+\frac{1}{\xi_{2}\left(\eta_{2}-1\right)}$$

**Lemma** **2.** *With the fractional derivative such as ${}_{a}D_{t}^{\alpha_{1}}f\left(t\right)=\frac{1}{\Gamma (1-\alpha_{1})}\frac{d}{dt}\int_{a}^{t}\frac{f(\tau )}{{\left(t-\tau \right)}^{\alpha_{1}}}d\tau$ with f(t)∈R, $0\leq \alpha_{1}<1$, and its sign function, then, for the fractional derivative of the sign function [45], one obtains ${}_{a}D_{t}^{\alpha_{1}}sign\left(f\left(t\right)\right)\left\{\begin{array}{cc} >0 & if\;\;f(t)>0,\;t>0\\ <0 & if\;\;f(t)<0,\;t>0 \end{array}\right.$.*

## 3. Fractional-Order Fixed-Time Non-Singular Terminal Sliding Control Design

This part begins with an introduction to the dynamics of the robot manipulator and continues with a study of the characteristics of a fractional-order non-singular fixed-time sliding surface and the development of a control design called FoFxNTSM. In addition to this, a study of the suggested FoFxNTSM’s stability using the Lyapunov theorem is presented.

The following is a description of the dynamic equation of the n−DOF robotic manipulator [46].

(5) $$M\left(q\right)\ddot{q}+C\left(q,\dot{q}\right)\dot{q}+G\left(q\right)=\tau \left(t\right)+\tau_{f}\left(t\right)+\tau_{d}\left(t\right)$$

where $q\;\in \;R{}^{n}$ is the joints position, $\dot{q}\;\in \;R{}^{n}$ is the joint velocity, and $\ddot{q}\;\in \;R{}^{n}$ is the joint acceleration. $M\left(q\right)\;\in \;R{}^{n\times n}$ represents the inertia matrix and satisfies that $m_{1}\left(M\left(q\right)\right)\leq ∥M(q)∥\leq m_{2}\left(M\left(q\right)\right)$, with $m_{1}$ and $m_{2}$ illustrating the positive min and the max eigenvalues of the matrix M(q). $C\left(q,\dot{q}\right)\in \;R{}^{n\times n}$ denotes the coriolis, centripetal, and friction forces matrix; $G\left(q\right)\in \;R{}^{n}$ is the gravitational vector. $\tau_{f}\in \;R{}^{n}$ is system’s uncertainty, $\tau_{d}\in \;R{}^{n}$ is a representation of the unknown external disturbance, $\tau \left(t\right)\in \;R{}^{n}$ is the input torque at the joints.

The dynamic Equation (5) can be rewritten as

(6) $$\ddot{q}=M^{-1}\left(q\right)\tau -M^{-1}\left(q\right)\left[C\left(q,\dot{q}\right)\dot{q}+G\left(q\right)\right]+ℑ\left(q,\dot{q},\ddot{q},\tau_{d}\right)$$

where $ℑ\left(q,\dot{q},\ddot{q},\tau_{d}\right)=M^{-1}\left(q\right)[\tau_{d}\left(t\right)+\tau_{f}\left(t\right)]$ represents the uncertainties and external disturbances.

Using Equation (6), the trajectory tracking error can be expressed as

(7) $$\ddot{\varepsilon }=M^{-1}\left(q\right)\tau +\partial \left(q,\dot{q}\right)+ℑ\left(q,\dot{q},\ddot{q},\tau_{d}\right)$$

where $\partial \left(q,\dot{q}\right)=-M^{-1}\left(q\right)\left[C\left(q,\dot{q}\right)\dot{q}+G\left(q\right)\right]-{\ddot{q}}_{d}$ denotes the known system dynamics. The tracking error is represented by the equation $\varepsilon =q-q_{d}$, where *q* represents the actual position vectors and $q_{d}$ represents the desired position vectors.

**Assumption** **1.** Conditional bounds on the uncertainty and external disturbance are expressed by (8), which is shown below:

(8) $$∥ℑ(q,\dot{q},\ddot{q},\tau_{d})∥\leq \iota_{1}+\iota_{2}∥q∥+\iota_{3}{∥\dot{q}∥}^{2}$$

where $\iota_{1}$, $\iota_{2}$, and $\iota_{3}$ are unknown constants of the uncertainties’ and disturbances’ upper bounds.

### 3.1. FoFxNTSM Surface

The aforementioned techniques served as inspiration for the development of the fractional-order non-singular terminal sliding mode control, which can be built to provide the robust and precise tracking performance of the n−DOF robotic manipulators in a fixed time. Therefore, based on the features of fractional-order calculus, the proposed sliding surface is given as

(9) $$s\left(t\right)=\dot{\varepsilon }\left(t\right)+\delta_{1}\sqrt[{1/\beta_{1}}]{\left|\varepsilon \right|}sign\left(\varepsilon \right)+\delta_{2}\sqrt[{1/\beta_{2}}]{\left|\varepsilon \right|}sign\left(\varepsilon \right)+\delta_{3}D^{\alpha -1}\left[\left|\varepsilon \right|sign\left(\varepsilon \right)\right]$$

where $s\left(t\right)\in R{}^{n}$ is the sliding surface, and $\delta_{1}\in R{}^{+}$ and $\delta_{2}\in R{}^{+}$ are positive constants. To be more specific, $\beta_{1}$ and $\beta_{2}$ are the set of constants, such that $0<\beta_{1}<1$, $1<\beta_{2}$, and 0<α<1.

(10) $$\dot{s}\left(t\right)=\ddot{\varepsilon }\left(t\right)+\beta_{1}\delta_{1}{\left|\varepsilon \right|}^{\beta_{1}-1}\dot{\varepsilon }+\beta_{2}\delta_{2}{\left|\varepsilon \right|}^{\beta_{2}-1}\dot{\varepsilon }+\delta_{3}D^{\alpha }\left[\left|\varepsilon \right|sign\left(\varepsilon \right)\right]$$

(11) $$\begin{array}{c} \dot{s}\left(t\right)=M^{-1}\left(q\right)\tau +\partial \left(q,\dot{q}\right)+ℑ\left(q,\dot{q},\ddot{q},\tau_{d}\right)\\ +\delta_{1}K\left(\varepsilon \right)\dot{\varepsilon }+\beta_{2}\delta_{2}{\left|\varepsilon \right|}^{\beta_{2}-1}\dot{\varepsilon }+\delta_{3}D^{\alpha }\left[\left|\varepsilon \right|sign\left(\varepsilon \right)\right] \end{array}$$

where $K\left(\varepsilon \right)=\left\{\begin{array}{cc} \beta_{1}{\left|\varepsilon \right|}^{\beta_{1}-1} & if\;\;\varepsilon \neq 0\\ 0 & if\;\;\varepsilon =0 \end{array}\right.$.

Now that the construction of the sliding manifold is complete, the robust performance against uncertainty and external disturbances is achieved using the proposed FoFxNTSM control design for n−DOF robotic manipulators.

Throughout the course of the sliding mode, when s(t)=0, the following dynamics can be derived from (9) as

(12) $$\dot{\varepsilon }\left(t\right)=-\delta_{1}\sqrt[{1/\beta_{1}}]{\left|\varepsilon \right|}sign\left(\varepsilon \right)-\delta_{2}\sqrt[{1/\beta_{2}}]{\left|\varepsilon \right|}sign\left(\varepsilon \right)-\delta_{3}D^{\alpha -1}\left[\left|\varepsilon \right|sign\left(\varepsilon \right)\right]$$

The Lyapunov function is defined as follows

(13) $$V_{1}\left(t\right)=0.5\varepsilon {\left(t\right)}^{T}\varepsilon \left(t\right)$$

With (13), the ${\dot{V}}_{1}\left(t\right)$ can be computed as

(14) $${\dot{V}}_{1}\left(t\right)=\varepsilon {\left(t\right)}^{T}\dot{\varepsilon }\left(t\right)=\varepsilon {\left(t\right)}^{T}\left[-\delta_{1}\sqrt[{1/\beta_{1}}]{\left|\varepsilon \right|}sign\left(\varepsilon \right)-\delta_{2}\sqrt[{1/\beta_{2}}]{\left|\varepsilon \right|}sign\left(\varepsilon \right)-\delta_{3}D^{\alpha -1}\left[\left|\varepsilon \right|sign\left(\varepsilon \right)\right]\right]$$

By simplifying (14), one has

(15) $${\dot{V}}_{1}\left(t\right)=-\delta_{1}\varepsilon {\left(t\right)}^{T}\sqrt[{1/\beta_{1}}]{\left|\varepsilon \right|}sign\left(\varepsilon \right)-\delta_{2}\varepsilon {\left(t\right)}^{T}\sqrt[{1/\beta_{2}}]{\left|\varepsilon \right|}sign\left(\varepsilon \right)-\delta_{3}{\left|\varepsilon (t)\right|}^{T}D^{\alpha -1}\left[\left|\varepsilon \right|sign^{2}\left(\varepsilon \right)\right]$$

(16) $${\dot{V}}_{1}\left(t\right)\leq -\delta_{1}{∥\varepsilon ∥}^{\beta_{1}+1}-\delta_{2}{∥\varepsilon ∥}^{\beta_{2}+1}$$

(17) $${\dot{V}}_{1}\left(t\right)\leq -2^{\frac{\beta_{1}+1}{2}}\delta_{1}V_{1}^{\frac{\beta_{1}+1}{2}}-2^{\frac{\beta_{2}+1}{2}}\delta_{2}V_{1}^{\frac{\beta_{2}+1}{2}}$$

In accordance with Lemma 1, the sliding surface (9) converges to zero in a fixed time, and the amount of time it takes to get there is bounded by

(18) $$\begin{array}{c} T_{1}=\frac{1}{2^{\frac{\beta_{1}+1}{2}}\delta_{1}\left(1-\frac{\beta_{1}+1}{2}\right)}+\frac{1}{2^{\frac{\beta_{2}+1}{2}}\delta_{2}\left(\frac{\beta_{2}+1}{2}-1\right)}\\ \;\;\;=\frac{2}{2^{\frac{\beta_{1}+1}{2}}\delta_{1}\left(1-\beta_{1}\right)}+\frac{2}{2^{\frac{\beta_{2}+1}{2}}\delta_{2}\left(\beta_{2}-1\right)} \end{array}$$

### 3.2. FoFxNTSM Control Design

For the purpose of controlling a robotic manipulator in the presence of known bounded uncertainties and external disturbances, the FoFxNTSM control law can be designed as follows

(19) $$\tau \left(t\right)=\tau_{nm}\left(t\right)+\tau_{sw}\left(t\right)$$

where $\tau_{nm}\left(t\right)$ refers to the control input that is employed in the control of the known dynamics and $\tau_{sw}\left(t\right)$ refers to the control input that is utilized to deal with uncertain dynamics.

(20) $$\tau_{nm}=-M\left(q\right)\left\{\begin{array}{c} \partial \left(q,\dot{q}\right)+\delta_{1}K\left(\varepsilon \right)\dot{\varepsilon }+\beta_{2}\delta_{2}{\left|\varepsilon \right|}^{\beta_{2}-1}\dot{\varepsilon }+\delta_{3}D^{\alpha }\left[\left|\varepsilon \right|sign\left(\varepsilon \right)\right] \end{array}\right\}$$

(21) $$\tau_{sw}=-M\left(q\right)\left\{\begin{array}{c} \left(\iota_{1}+\iota_{2}∥q∥+\iota_{3}{∥\dot{q}∥}^{2}\right)sign\left(s\right)\\ +\delta_{4}\sqrt[{1/\varsigma_{1}}]{\left|s\right|}sign\left(s\right)+\delta_{5}\sqrt[{1/\varsigma_{2}}]{\left|s\right|}sign\left(s\right)+\delta_{6}D^{\alpha_{1}}sign\left(s\right) \end{array}\right\}$$

where $\delta_{4}\in R{}^{+}$, $\delta_{5}\in R{}^{+}$ and $\delta_{6}\in R{}^{+}$ are positive constants, and $\varsigma_{1}$ and $\varsigma_{2}$ are constants, such that $0<\varsigma_{1}<1$, $1<\varsigma_{2}$ and $0\leq \alpha_{1}<1$, respectively.

### 3.3. Stability Analysis

The Lyapunov theorem is applied in this subsection to establish the closed-loop system stability.

**Theorem** **1.** *Considering the described robotic manipulator (5), the suggested sliding manifold (9) and the designed FoFxNTSM controller (19) enable the intended angular position of the uncertain robotic manipulator to converge in a fixed amount of time with condition (8).*

**Proof.**  The Lyapunov function is considered as follows

(22) $$V_{2}\left(t\right)=0.5s{\left(t\right)}^{T}s\left(t\right)$$

where ${\dot{V}}_{2}\left(t\right)$ can be computed as

(23) $${\dot{V}}_{2}\left(t\right)=s{\left(t\right)}^{T}\dot{s}\left(t\right)$$

With $\dot{s}\left(t\right)$ from (10) substituted into Equation (23), one obtains

(24) $${\dot{V}}_{2}\left(t\right)=s{\left(t\right)}^{T}\left[\ddot{\varepsilon }\left(t\right)+\delta_{1}K\left(\varepsilon \right)\dot{\varepsilon }+\beta_{2}\delta_{2}{\left|\varepsilon \right|}^{\beta_{2}-1}\dot{\varepsilon }+\delta_{3}D^{\alpha }\left[\left|\varepsilon \right|sign\left(\varepsilon \right)\right]\right]$$

By substituting $\ddot{\varepsilon }\left(t\right)$ from (7) in (24), one obtains

(25) $${\dot{V}}_{2}\left(t\right)=s{\left(t\right)}^{T}\left\{\begin{array}{c} M^{-1}\left(q\right)\tau +\partial \left(q,\dot{q}\right)+ℑ\left(q,\dot{q},\ddot{q},\tau_{d}\right)\\ +\delta_{1}K\left(\varepsilon \right)\dot{\varepsilon }+\beta_{2}\delta_{2}{\left|\varepsilon \right|}^{\beta_{2}-1}\dot{\varepsilon }+\delta_{3}D^{\alpha }\left[\left|\varepsilon \right|sign\left(\varepsilon \right)\right] \end{array}\right\}$$

By substituting τ(t) from (19) in (25), one has

(26) $${\dot{V}}_{2}\left(t\right)=s{\left(t\right)}^{T}\left[\begin{array}{c} -\left\{\begin{array}{c} \left(\iota_{1}+\iota_{2}∥q∥+\iota_{3}{∥\dot{q}∥}^{2}\right)sign\left(s\right)+\partial \left(q,\dot{q}\right)\\ +\delta_{1}K\left(\varepsilon \right)\dot{\varepsilon }+\beta_{2}\delta_{2}{\left|\varepsilon \right|}^{\beta_{2}-1}\dot{\varepsilon }+\delta_{3}D^{\alpha }\left[\left|\varepsilon \right|sign\left(\varepsilon \right)\right]\\ +\delta_{4}\sqrt[{1/\varsigma_{1}}]{\left|s\right|}sign\left(s\right)+\delta_{5}\sqrt[{1/\varsigma_{2}}]{\left|s\right|}sign\left(s\right)+\delta_{6}D^{\alpha_{1}}sign\left(s\right) \end{array}\right\}\\ +\partial \left(q,\dot{q}\right)+ℑ\left(q,\dot{q},\ddot{q},\tau_{d}\right)+\delta_{1}K\left(\varepsilon \right)\dot{\varepsilon }+\beta_{2}\delta_{2}{\left|\varepsilon \right|}^{\beta_{2}-1}\dot{\varepsilon }+\delta_{3}D^{\alpha }\left[\left|\varepsilon \right|sign\left(\varepsilon \right)\right] \end{array}\right]$$

The simplification of (26) yields

(27) $${\dot{V}}_{2}\left(t\right)=s{\left(t\right)}^{T}\left[\begin{array}{c} -\left\{\begin{array}{c} \left(\iota_{1}+\iota_{2}∥q∥+\iota_{3}{∥\dot{q}∥}^{2}\right)sign\left(s\right)+\delta_{4}\sqrt[{1/\varsigma_{1}}]{\left|s\right|}sign\left(s\right)\\ +\delta_{5}\sqrt[{1/\varsigma_{2}}]{\left|s\right|}sign\left(s\right)+\delta_{6}D^{\alpha_{1}}sign\left(s\right) \end{array}\right\}\\ +ℑ(q,\dot{q},\ddot{q},\tau_{d}) \end{array}\right]$$

According to Assumption 1 and Lemma 2, one can easily obtain

(28) $${\dot{V}}_{2}\left(t\right)\leq -\delta_{4}{∥s∥}^{\varsigma_{1}+1}-\delta_{5}{∥s∥}^{\varsigma_{2}+1}$$

and (28) can be rewritten as

(29) $${\dot{V}}_{2}\left(t\right)\leq -2^{\frac{\varsigma_{1}+1}{2}}\delta_{4}V_{2}{\left(t\right)}^{\frac{\varsigma_{1}+1}{2}}-2^{\frac{\varsigma_{2}+1}{2}}\delta_{5}V_{2}{\left(t\right)}^{\frac{\varsigma_{2}+1}{2}}$$

Therefore, the trajectory of the system reaches s(t) in a fixed time. In accordance with Lemma 1, the time required for convergence can be expressed as

(30) $$T_{2}=\frac{1}{2^{\frac{\varsigma_{1}+1}{2}}\delta_{4}\left(1-\frac{\varsigma_{1}+1}{2}\right)}+\frac{1}{2^{\frac{\varsigma_{2}+1}{2}}\delta_{5}\left(\frac{\varsigma_{2}+1}{2}-1\right)}$$

Using relation $T_{s1}=T_{1}+T_{2}$, the settling time $T_{s1}$ can be formulated as

(31) $$T_{s1}=\frac{2}{2^{\frac{\beta_{1}+1}{2}}\delta_{1}\left(1-\beta_{1}\right)}+\frac{2}{2^{\frac{\beta_{2}+1}{2}}\delta_{2}\left(\beta_{2}-1\right)}+\frac{2}{2^{\frac{\varsigma_{1}+1}{2}}\delta_{4}\left(1-\varsigma_{1}\right)}+\frac{2}{2^{\frac{\varsigma_{2}+1}{2}}\delta_{5}\left(\varsigma_{2}-1\right)}$$

As a result, it can be deduced from (31) that the suggested scheme is a fixed-time control scheme. □

## 4. Adaptive FoFxNTSM Control Design

The following describes how the control input utilizing an adaptive method is devised to account for the unknown dynamics and external disturbances.

(32) $$\tau \left(t\right)=\tau_{ad}\left(t\right)$$

(33) $$\tau_{ad}\left(t\right)=-M{\left(q\right)}^{}\left\{\begin{array}{c} \left({\hat{\iota }}_{1}+{\hat{\iota }}_{2}∥q∥+{\hat{\iota }}_{3}{∥\dot{q}∥}^{2}\right)sign\left(s\right)+\partial \left(q,\dot{q}\right)\\ +\delta_{1}K\left(\varepsilon \right)\dot{\varepsilon }+\beta_{2}\delta_{2}{\left|\varepsilon \right|}^{\beta_{2}-1}\dot{\varepsilon }+\delta_{3}D^{\alpha }\left[\left|\varepsilon \right|sign\left(\varepsilon \right)\right]\\ +\delta_{4}\sqrt[{1/\varsigma_{1}}]{\left|s\right|}sign\left(s\right)+\delta_{5}\sqrt[{1/\varsigma_{2}}]{\left|s\right|}sign\left(s\right)+\delta_{6}D^{\alpha_{1}}sign\left(s\right) \end{array}\right\}$$

where $\;{\hat{\iota }}_{1}$, $\;{\hat{\iota }}_{2}$, and $\;{\hat{\iota }}_{3}$ denote the estimation variable of $\iota_{1},\;\;\iota_{2}$, and $\iota_{3}$, respectively.

**Figure 1:**
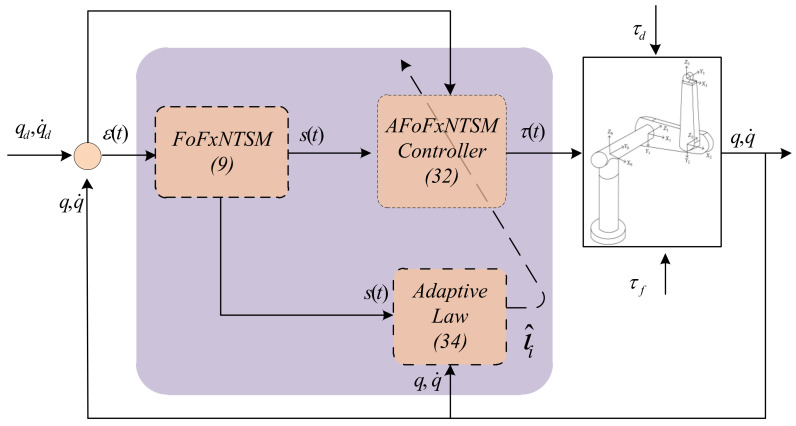
Control model of proposed scheme.

To compensate for unknown dynamics, the adaptive laws are proposed. In addition, the dead-zone method is applied to avoid the parameter drifting problem; thus, the adaptive laws are given as

(34) $${\dot{\hat{\iota }}}_{i}=\left\{\begin{array}{cc} \gamma_{i}∥s∥\Delta \varpi & if\;\;∥s∥>\varpi \\ 0 & if\;\;∥s∥\leq \varpi \end{array}\;\;\;\;\;\;\;\&\;\;\;\;\;\;\;i=1,2,3\right.$$

where $\Delta \varpi =\left[1,\;\;∥q∥,\;\;{∥\dot{q}∥}^{2}\right]$, ϖ>0 denotes the size of the dead zone, and $\gamma_{1},\;\gamma_{2}\;$, and $\;\gamma_{3}>0$ are constants. The proposed model is given in Figure 1.

Compensating for the upper bounds of the unknown dynamics is dealt with the use of (34). Therefore, the AFoFxNTSM technique is what ultimately determines the tracking performance of the uncertain robot manipulators under disturbances.

**Theorem** **2.** *Considering the given robotic manipulator (5) and its susceptibility to issues such as uncertainty and external disturbances, accordingly, the desired angular position of the robotic manipulator converges in a fixed time with the condition of Assumption 1, thanks to the suggested sliding surface (9), AFoFxNTSM control input (32), and adaptive laws (34).*

**Proof.**  The following Lyapunov candidate is selected as

(35) $$V_{3}\left(t\right)=0.5s{\left(t\right)}^{T}s\left(t\right)+\frac{0.5}{\gamma_{1}}{\tilde{\iota }}_{1}^{2}+\frac{0.5}{\gamma_{2}}{\tilde{\iota }}_{2}^{2}+\frac{0.5}{\gamma_{3}}{\tilde{\iota }}_{3}^{2}$$

where ${\tilde{\iota }}_{1}={\hat{\iota }}_{1}-\iota_{1},\;\;{\tilde{\iota }}_{2}={\hat{\iota }}_{2}-\iota_{2},\;{\tilde{\iota }}_{3}={\hat{\iota }}_{3}-\iota_{3}$ are estimation errors.

${\dot{V}}_{3}\left(t\right)$ can be expressed as

(36) $${\dot{V}}_{3}\left(t\right)=s{\left(t\right)}^{T}\dot{s}\left(t\right)+\frac{1}{\gamma_{1}}{\tilde{\iota }}_{1}^{}{\dot{\hat{\iota }}}_{1}^{}+\frac{1}{\gamma_{2}}{\tilde{\iota }}_{2}^{}{\dot{\hat{\iota }}}_{2}^{}+\frac{1}{\gamma_{3}}{\tilde{\iota }}_{3}^{}{\dot{\hat{\iota }}}_{3}^{}$$

With the substitution of $\dot{s}\left(t\right)$ from (11) into (36), one can obtain

(37) $$\begin{array}{c} {\dot{V}}_{3}\left(t\right)=s{\left(t\right)}^{T}\left\{M^{-1}\left(q\right)\tau +\partial \left(q,\dot{q}\right)+ℑ\left(q,\dot{q},\ddot{q},\tau_{d}\right)+\delta_{1}K\left(\varepsilon \right)\dot{\varepsilon }+\beta_{2}\delta_{2}{\left|\varepsilon \right|}^{\beta_{2}-1}\dot{\varepsilon }+\delta_{3}D^{\alpha }\left[\left|\varepsilon \right|sign\left(\varepsilon \right)\right]\right\}\\ +\frac{1}{\gamma_{1}}{\tilde{\iota }}_{1}^{}{\dot{\hat{\iota }}}_{1}^{}+\frac{1}{\gamma_{2}}{\tilde{\iota }}_{2}^{}{\dot{\hat{\iota }}}_{2}^{}+\frac{1}{\gamma_{3}}{\tilde{\iota }}_{3}^{}{\dot{\hat{\iota }}}_{3}^{} \end{array}$$

With the substitution of τ(t) from (32) into (37), one can obtain

(38) $$\begin{array}{c} {\dot{V}}_{3}\left(t\right)=s{\left(t\right)}^{T}\left[\begin{array}{c} -\left\{\begin{array}{c} \left({\hat{\iota }}_{1}+{\hat{\iota }}_{2}∥q∥+{\hat{\iota }}_{3}{∥\dot{q}∥}^{2}\right)sign\left(s\right)+\partial \left(q,\dot{q}\right)\\ +\delta_{1}K\left(\varepsilon \right)\dot{\varepsilon }+\beta_{2}\delta_{2}{\left|\varepsilon \right|}^{\beta_{2}-1}\dot{\varepsilon }\\ +\delta_{3}D^{\alpha }\left[\left|\varepsilon \right|sign\left(\varepsilon \right)\right]+\delta_{4}\sqrt[{1/\varsigma_{1}}]{\left|s\right|}sign\left(s\right)\\ +\delta_{5}\sqrt[{1/\varsigma_{2}}]{\left|s\right|}sign\left(s\right)+\delta_{6}D^{\alpha_{1}}sign\left(s\right) \end{array}\right\}\\ +\partial \left(q,\dot{q}\right)+ℑ\left(q,\dot{q},\ddot{q},\tau_{d}\right)+\delta_{1}K\left(\varepsilon \right)\dot{\varepsilon }\\ +\beta_{2}\delta_{2}{\left|\varepsilon \right|}^{\beta_{2}-1}\dot{\varepsilon }+\delta_{3}D^{\alpha }\left[\left|\varepsilon \right|sign\left(\varepsilon \right)\right] \end{array}\right]\\ +\frac{1}{\gamma_{1}}{\tilde{\iota }}_{1}^{}{\dot{\hat{\iota }}}_{1}^{}+\frac{1}{\gamma_{2}}{\tilde{\iota }}_{2}^{}{\dot{\hat{\iota }}}_{2}^{}+\frac{1}{\gamma_{3}}{\tilde{\iota }}_{3}^{}{\dot{\hat{\iota }}}_{3}^{} \end{array}$$

Simplifying (38) yields

(39) $$\begin{array}{c} {\dot{V}}_{3}\left(t\right)=s{\left(t\right)}^{T}\left[\begin{array}{c} -\left\{\begin{array}{c} \left({\hat{\iota }}_{1}+{\hat{\iota }}_{2}∥q∥+{\hat{\iota }}_{3}{∥\dot{q}∥}^{2}\right)sign\left(s\right)+\delta_{4}\sqrt[{1/\varsigma_{1}}]{\left|s\right|}sign\left(s\right)\\ +\delta_{5}\sqrt[{1/\varsigma_{2}}]{\left|s\right|}sign\left(s\right)+\delta_{6}D^{\alpha_{1}}sign\left(s\right) \end{array}\right\}\\ +ℑ(q,\dot{q},\ddot{q},\tau_{d}) \end{array}\right]\\ +\frac{1}{\gamma_{1}}{\tilde{\iota }}_{1}^{}{\dot{\hat{\iota }}}_{1}^{}+\frac{1}{\gamma_{2}}{\tilde{\iota }}_{2}^{}{\dot{\hat{\iota }}}_{2}^{}+\frac{1}{\gamma_{3}}{\tilde{\iota }}_{3}^{}{\dot{\hat{\iota }}}_{3}^{} \end{array}$$

According to Lemma 2, (39) can be computed as

(40) $$\begin{array}{c} {\dot{V}}_{3}\left(t\right)\leq -\delta_{4}{∥s∥}^{\varsigma_{1}+1}-\delta_{5}{∥s∥}^{\varsigma_{2}+1}-{\hat{\iota }}_{1}∥s∥-{\hat{\iota }}_{2}∥q∥∥s∥-{\hat{\iota }}_{3}{∥\dot{q}∥}^{2}∥s∥+∥ℑ\left(q,\dot{q},\ddot{q},\tau_{d}\right)∥∥s∥\\ +\frac{1}{\gamma_{1}}{\tilde{\iota }}_{1}^{}{\dot{\hat{\iota }}}_{1}^{}+\frac{1}{\gamma_{2}}{\tilde{\iota }}_{2}^{}{\dot{\hat{\iota }}}_{2}^{}+\frac{1}{\gamma_{3}}{\tilde{\iota }}_{3}^{}{\dot{\hat{\iota }}}_{3}^{} \end{array}$$

Using Assumption 1 and the substitution of (34) into (40), one can obtain

(41) $${\dot{V}}_{3}\left(t\right)\leq -\delta_{4}{∥s∥}^{\varsigma_{1}+1}-\delta_{5}{∥s∥}^{\varsigma_{2}+1}$$

As a result, the robotic manipulator that is utilized for the purpose of precise trajectory tracking is only capable of maintaining its fixed-time stability under specific circumstances. As a consequence of this, the proof of stability is investigated in great detail.

Following that, the fixed settling time is calculated, and Equation (41) can be expressed as [47]

(42) $${\dot{V}}_{3}\left(t\right)\leq -\delta_{4}{\left\{2(V_{3}\left(t\right)-\Xi )\right\}}^{\frac{\varsigma_{1}+1}{2}}-\delta_{5}{\left\{2(V_{3}\left(t\right)-\Xi )\right\}}^{\frac{\varsigma_{2}+1}{2}}$$

where $\Xi =\frac{0.5}{\gamma_{1}}{\tilde{\iota }}_{1}^{2}+\frac{0.5}{\gamma_{2}}{\tilde{\iota }}_{2}^{2}+\frac{0.5}{\gamma_{3}}{\tilde{\iota }}_{3}^{2}$

(43) $${\dot{V}}_{3}\left(t\right)\leq -\delta_{4}2^{\frac{\varsigma_{1}+1}{2}}{\left\{V_{3}\left(t\right)-\Xi \right\}}^{\frac{\varsigma_{1}+1}{2}}-\delta_{5}2^{\frac{\varsigma_{2}+1}{2}}{\left\{V_{3}\left(t\right)-\Xi \right\}}^{\frac{\varsigma_{2}+1}{2}}$$

(44) $${\dot{V}}_{3}\left(t\right)\leq -\delta_{4}2^{\frac{\varsigma_{1}+1}{2}}{\left\{1-\frac{\Xi }{V_{3}\left(t\right)}\right\}}^{\frac{\varsigma_{1}+1}{2}}V_{3}{\left(t\right)}^{\frac{\varsigma_{1}+1}{2}}-\delta_{5}2^{\frac{\varsigma_{2}+1}{2}}{\left\{1-\frac{\Xi }{V_{3}\left(t\right)}\right\}}^{\frac{\varsigma_{2}+1}{2}}V_{3}{\left(t\right)}^{\frac{\varsigma_{2}+1}{2}}$$

Calculating the fixed time using Lemma 1 yields the following

(45) $$T_{3}=\frac{1}{p_{1}\left(1-\frac{\varsigma_{1}+1}{2}\right)}+\frac{1}{p_{2}\left(\frac{\varsigma_{2}+1}{2}-1\right)}=\frac{2}{p_{1}\left(1-\varsigma_{1}\right)}+\frac{2}{p_{2}\left(\varsigma_{2}-1\right)}$$

where $p_{1}=\delta_{4}2^{\frac{\varsigma_{1}+1}{2}}{\left\{1-\frac{\Xi }{V_{3}\left(t\right)}\right\}}^{\frac{\varsigma_{1}+1}{2}},\;\;\;\;$ and $\;p_{2}=\delta_{5}2^{\frac{\varsigma_{2}+1}{2}}{\left\{1-\frac{\Xi }{V_{3}\left(t\right)}\right\}}^{\frac{\varsigma_{2}+1}{2}}$. Calculating the settling time $T_{s2}$ using the relation $T_{s2}=T_{1}+T_{3}$ yields

(46) $$T_{s2}=\frac{2}{p_{1}\left(1-\varsigma_{1}\right)}+\frac{2}{p_{2}\left(\varsigma_{2}-1\right)}+\frac{2}{2^{\frac{\beta_{1}+1}{2}}\delta_{1}\left(1-\beta_{1}\right)}+\frac{2}{2^{\frac{\beta_{2}+1}{2}}\delta_{2}\left(\beta_{2}-1\right)}$$

The resulting state trajectory tends to zero in a fixed amount of time. □

**Remark** **1.** *When the proposed adaptive fractional-order fixed-time sliding mode control method is applied to the uncertain dynamics of the robotic system (5), which includes the fractional sliding surface (9), the proposed control input (32), and the adaptive laws (34), it is implied that the tracking error tends toward zero at a fixed time. The numerical simulation is provided in the following section.*

## 5. Simulation Results and Comparative Analyses

The PUMA 560 robotic manipulator is utilized to demonstrate the simulation performance in order to validate the AFoFxNTSM approach; its dynamics have been given in [48]. A 3−DOF of the PUMA 560 manipulator is employed, and it operates in an environment containing external disturbances and uncertainties. In order to show the great performance of AFoFxNTSM, two different scenarios, one with known dynamics and one with unknown uncertainties and disturbances, are described, and MATLAB/Simulink is used to simulate the proposed method. To demonstrate further the efficacy of the suggested strategy, a comparison is made with adaptive fractional-order non-singular terminal sliding mode control (ATDENTSM) [49]. Therefore, the planned trajectories, external disturbance, and uncertainty levels are given as:

$\begin{array}{c} q_{d}={\left[\;cos(t\pi /5)\;-1,\;\;cos(t\pi /5\;+\pi /2),\;\;cos(t\pi /5\;+\pi /2)-1\right]}^{T}\\ \tau_{f}={\left[0.5{\dot{q}}_{1}+sin\left(3q_{1}\right),\;\;1.3{\dot{q}}_{2}-1.8sin\left(2q_{2}\right),\;\;-1.8{\dot{q}}_{3}-2sin\left(q_{3}\right)\right]}^{T}\\ \tau_{d}={\left[20.5sin\left({\dot{q}}_{1}\right),\;\;21.1sin\left({\dot{q}}_{2}\right)\;,\;\;10.15sin\left({\dot{q}}_{3}\right)\right]}^{T} \end{array}$

**Figure 2:**
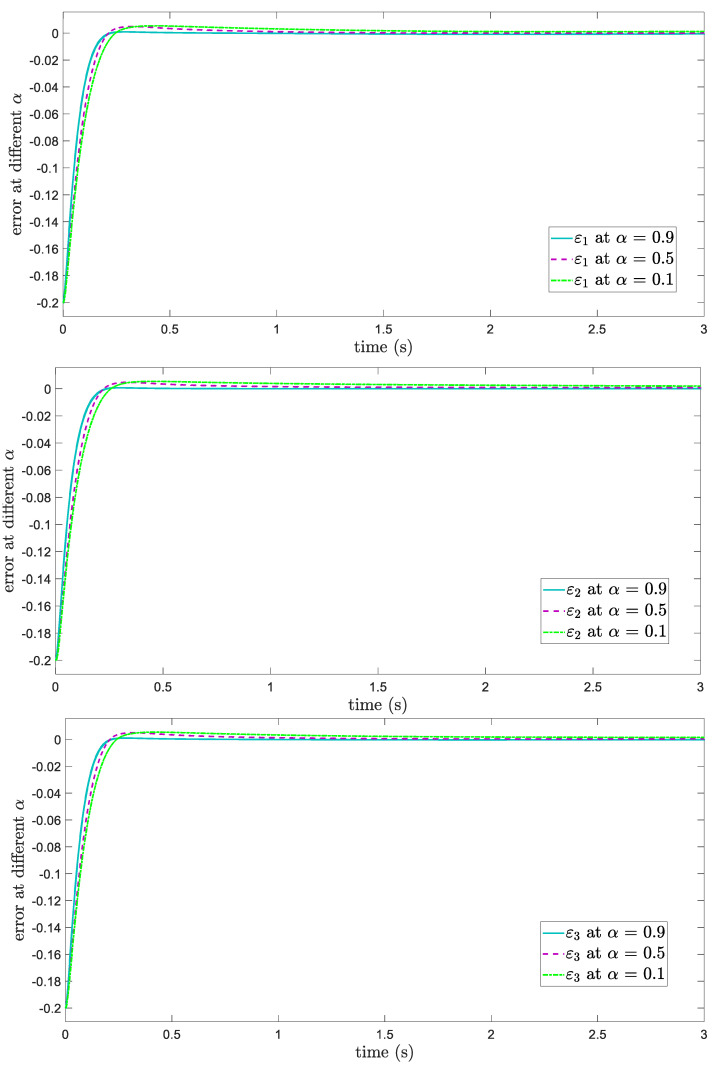
Tracking errors at different α values.

To select the suitable Fo value, the position tracking errors at different values of α are demonstrated in Figure 2.

As seen in Figure 2, setting α=0.9 is a simple way to achieve the best results. On the other hand, at α=0.1 and α=0.5, the desired trajectories are not achieved in terms of tracking errors.

### 5.1. Case 1: Comparison for Nominal Plant

In this subsection, the proposed FoFxNTSM approach is applied to the 3−DOF PUMA 560 robotic manipulator with known dynamics; however, external disturbances are not taken into consideration. For (9), the FoFxNTSM parameters are set to $\delta_{1}=6$, $\delta_{2}=6$, $\delta_{3}=6$, $\beta_{1}=0.8$, $\beta_{2}=1.9$, and α=0.9. The suitable parameters of (19) are set as $\delta_{4}=50$, $\delta_{5}=50$, $\delta_{6}=0.01$, $\alpha_{1}=0.1$, $\varsigma_{1}=0.7$, $\varsigma_{2}=1.5$, and ϖ= 0.1. The initial conditions of the joint positions are chosen as $q_{1}\left(0\right)=-0.2$, $q_{2}\left(0\right)=-0.2$, and $q_{3}\left(0\right)=-0.2$.

**Figure 3:**
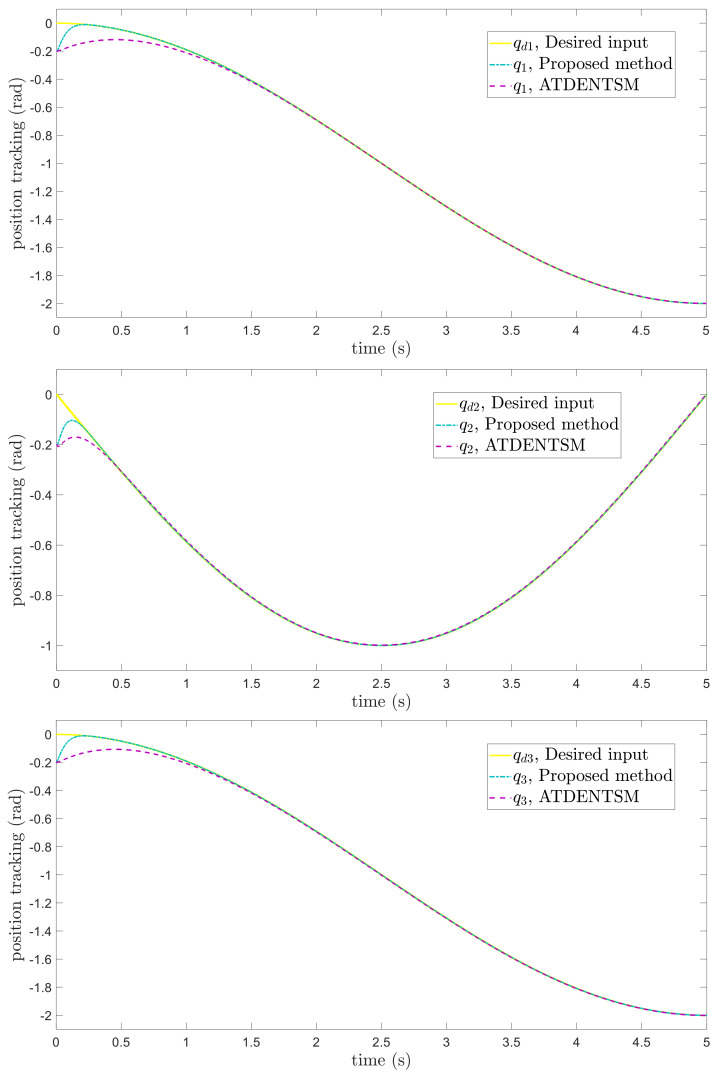
Position tracking.

**Figure 4:**
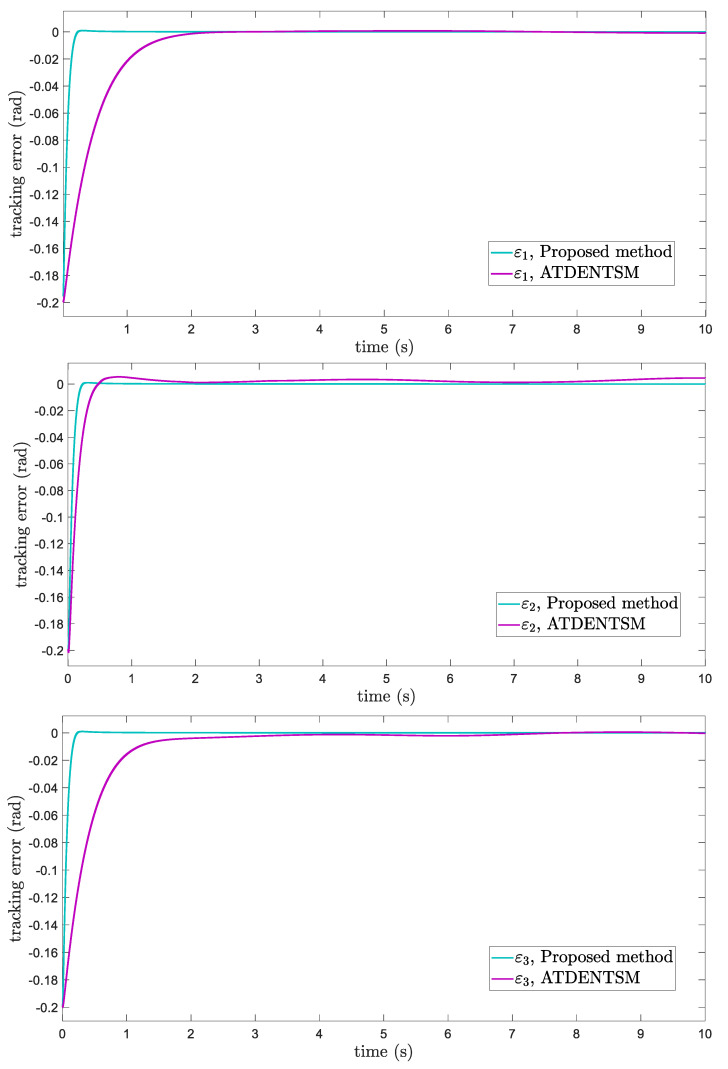
Tracking errors.

**Figure 5:**
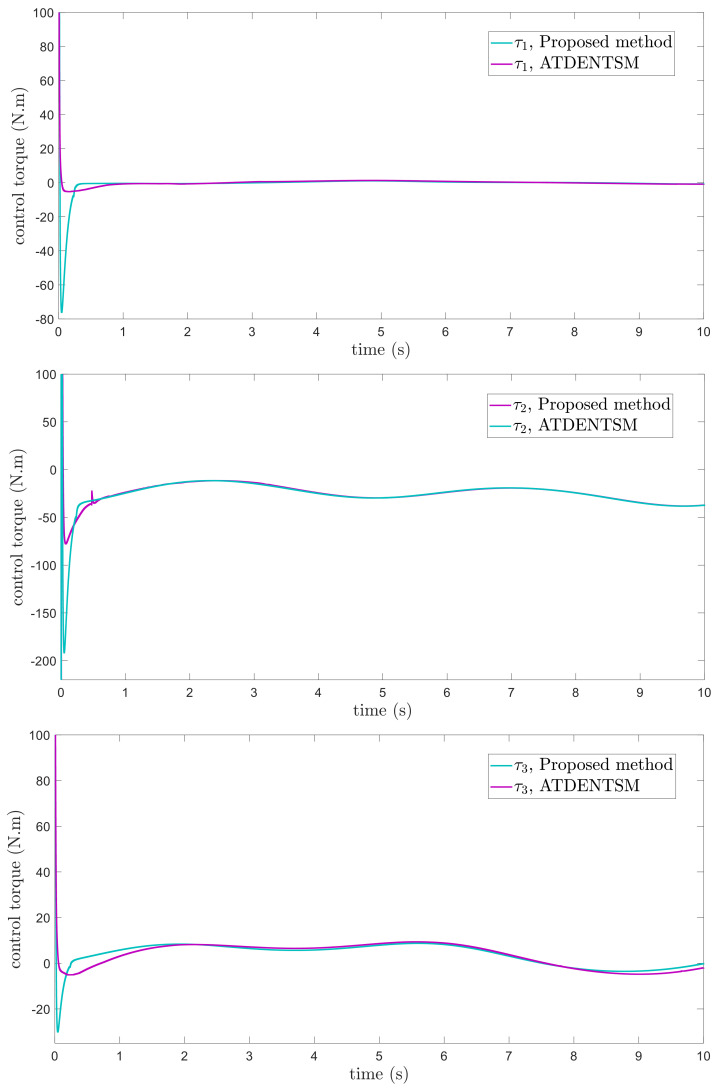
Control inputs.

**Figure 6:**
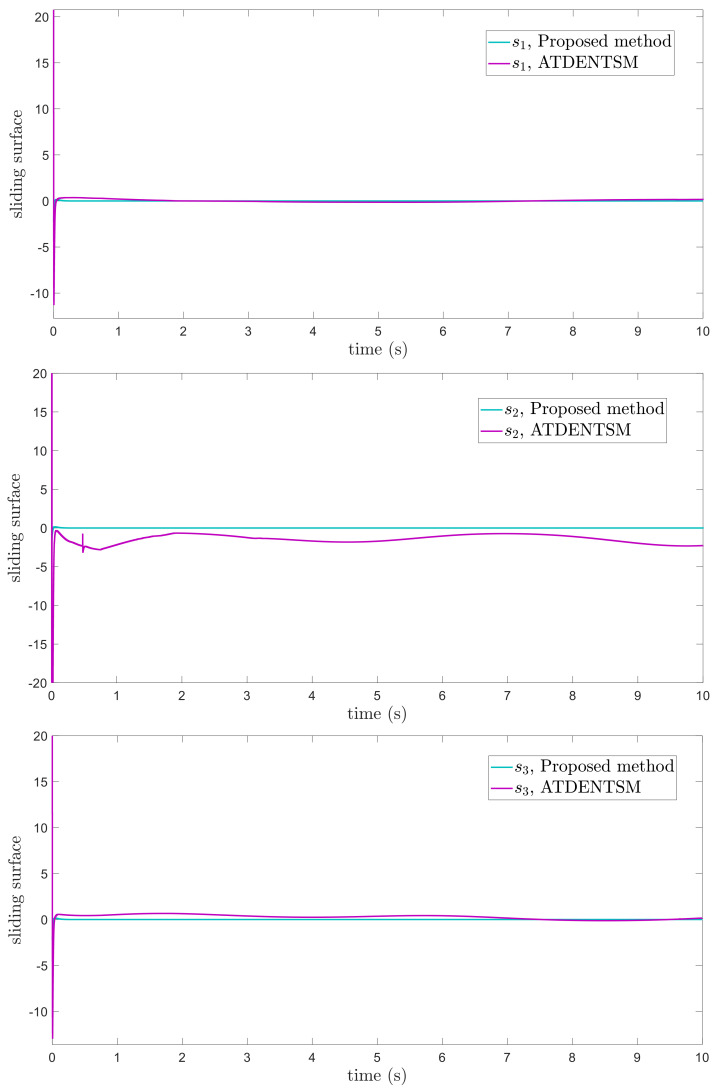
Sliding surfaces.

The comparative results of the proposed FoFxNTSM approach and ATDENTSM on 3−DOF robotic manipulators are depicted in Figure 3, Figure 4, Figure 5 and Figure 6, which show the joint’s position performance, its tracking errors, smooth control inputs, and sliding mode surfaces, respectively

The suggested FoFxNTSM scheme has improved performance and obtains small tracking errors, rapid convergence, and chatter-free control inputs. These advantages are achieved by taking into account the high tracking performance and robustness against the system’s known uncertainties.

### 5.2. Case 2: Comparison under Unknown Dynamics

**Figure 7:**
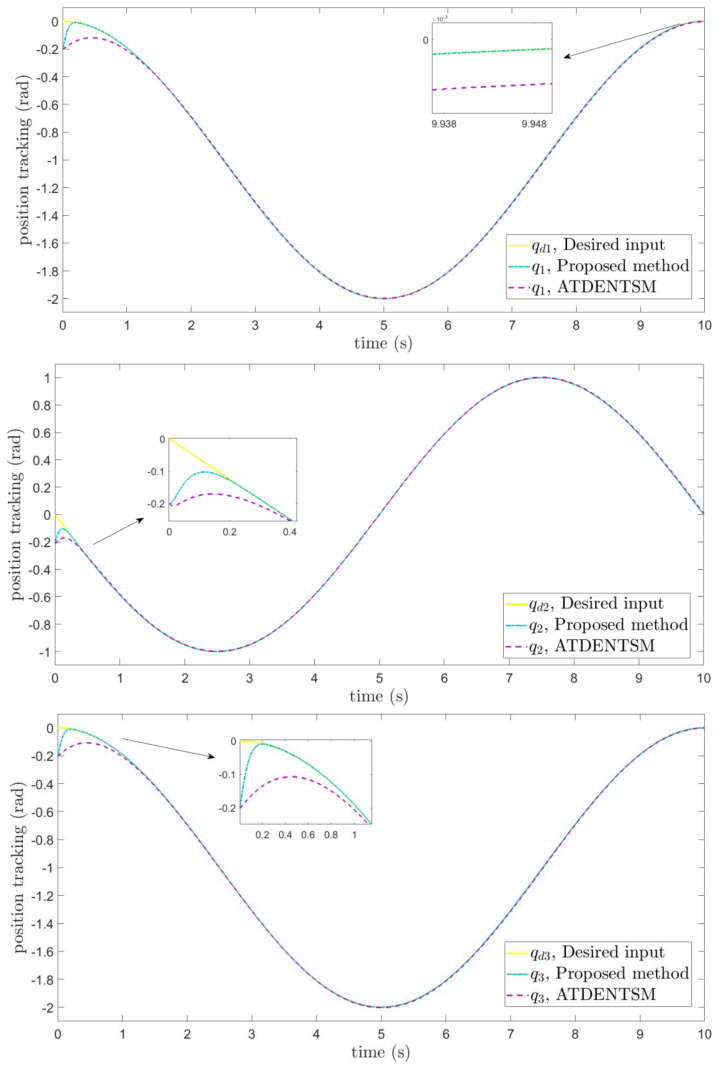
Position tracking method under uncertainties and disturbances.

**Figure 8:**
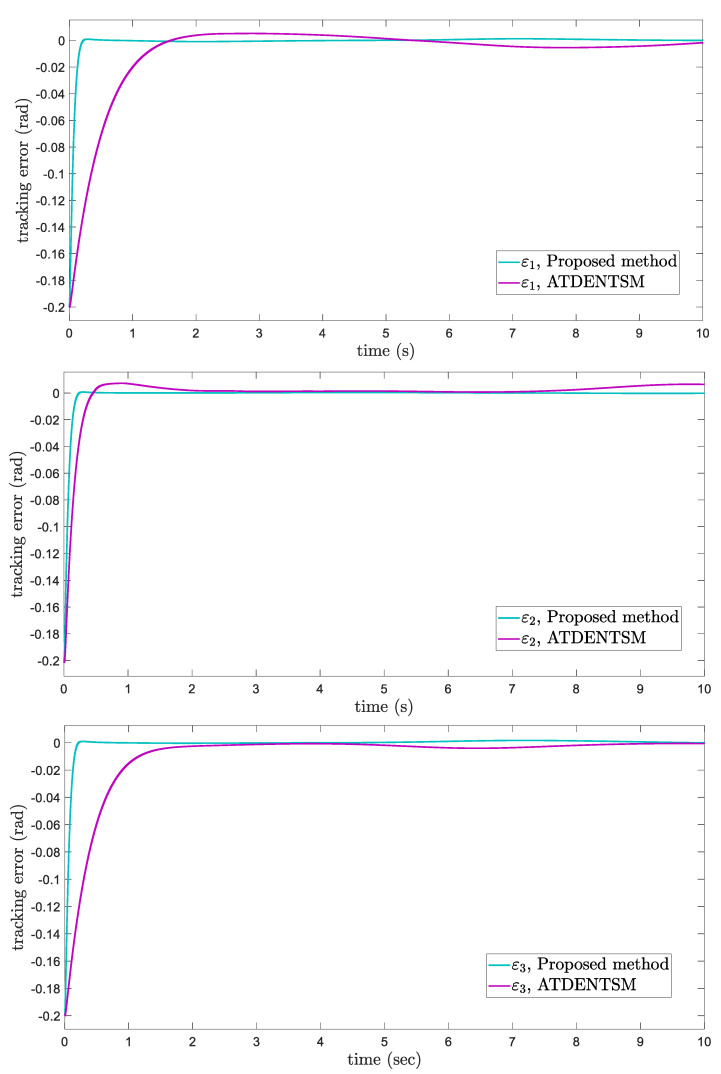
Tracking errors under uncertainties and disturbances.

**Figure 9:**
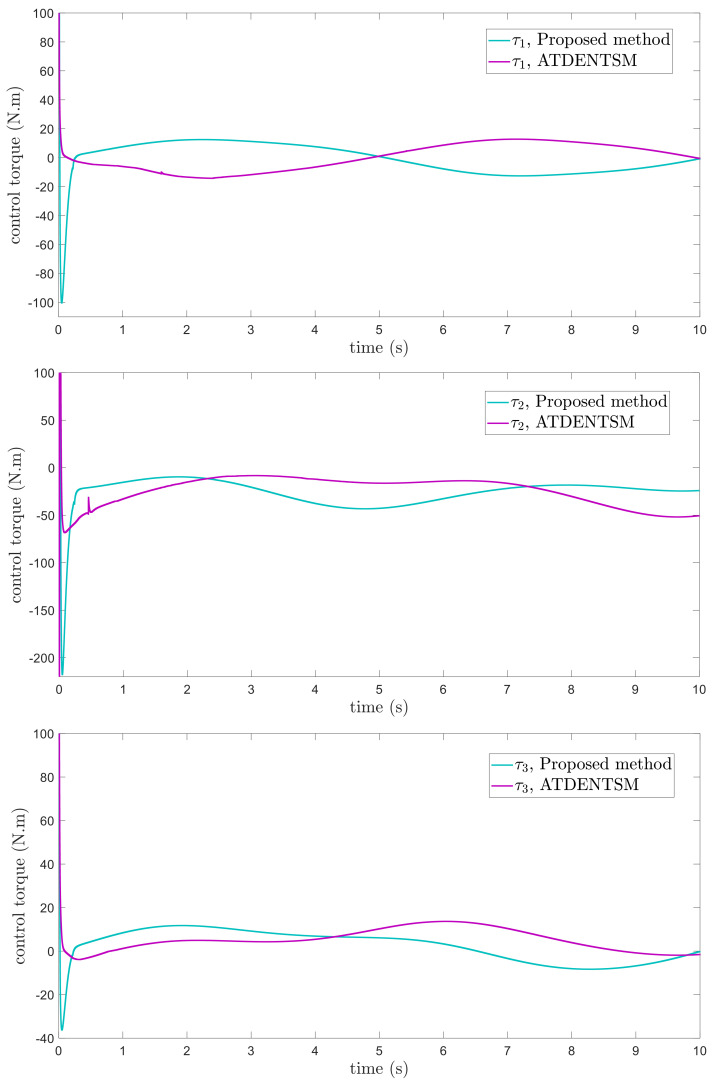
Control inputs under uncertainties and disturbances.

**Figure 10:**
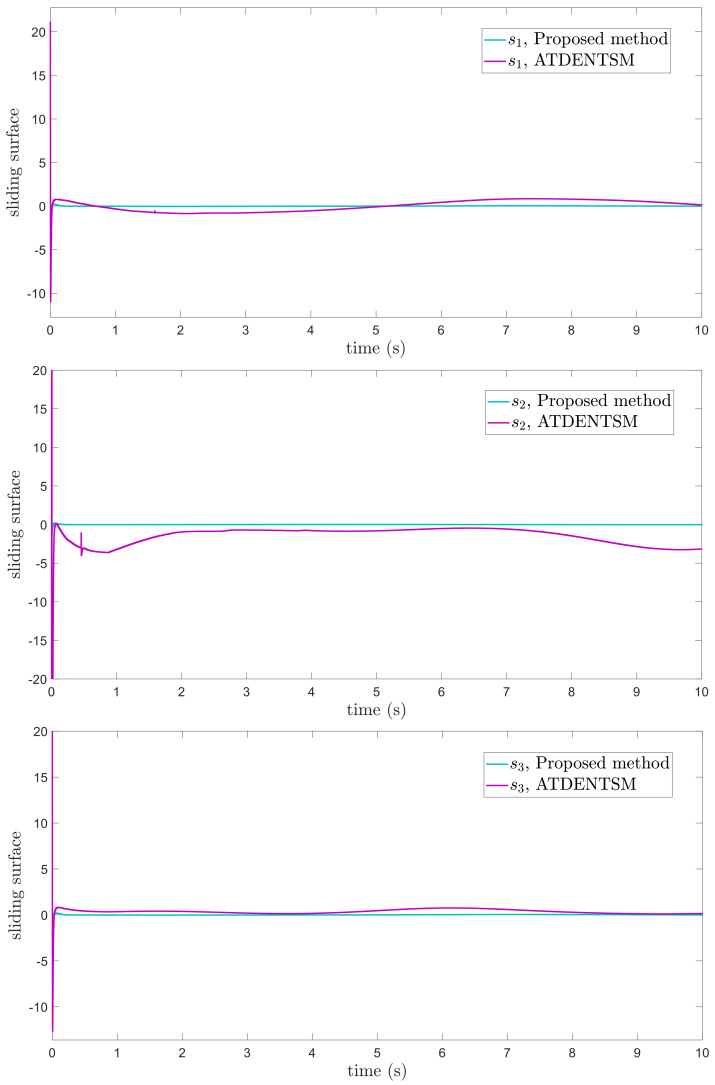
Sliding surfaces under uncertainties and disturbances.

**Figure 11:**
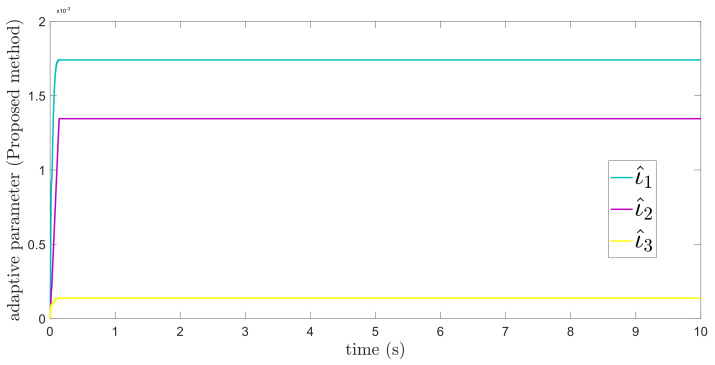
Adaptive parameters under uncertainties and disturbances—Proposed method.

**Figure 12:**
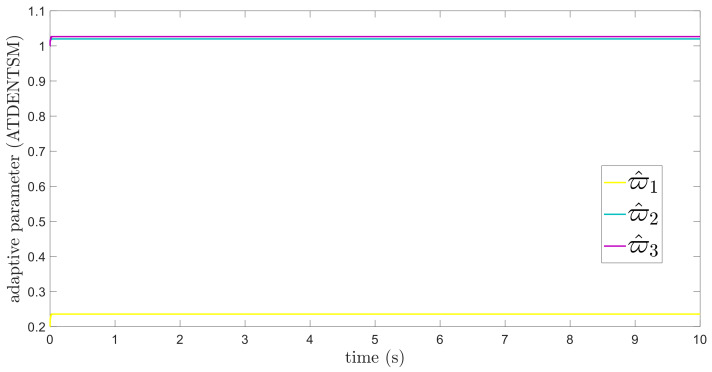
Adaptive parameters under uncertainties and disturbances—ATDENTSM.

In this subsection, the proposed adaptive technique with the FoFxNTSM method is used to control the dynamics of the 3−DOF robotic manipulator in the presence of unknown uncertainties, as well as external disturbances. The parameters of (32) are set such that they are identical to those of (19), and the parameters of (34) are set such that $\gamma_{1}=0.01$, $\gamma_{2}=0.01$, and $\gamma_{3}=0.01$. Figure 7, Figure 8, Figure 9 and Figure 10 present the results of comparing the proposed AFoFxNTSM scheme with ATDENTSM in terms of its performance in the face of unknown dynamics, as well as benchmark simulations of trajectories, control inputs, and sliding surfaces. Moreover, the adaptive parameter estimations of the unknown dynamics of AFoFxNTSM and ATDENTSM are given in Figure 11 and Figure 12, respectively.

The findings that are compared and obtained reveal that the AFoFxNTSM has an improved tracking performance, chatter-free control inputs, and adaptive estimation in the presence of unknown uncertainties and external disturbances. Figure 7, Figure 8, Figure 9, Figure 10, Figure 11 and Figure 12 make it abundantly clear that the proposed method, when subjected to uncertainties and external disturbances, yields a superior convergence and trajectory tracking performance, whereas the ATDENTSM method demonstrates significant angular position errors and is less robust when exposed to unknown dynamics.

## 6. Discussion

The discussion of the simulated results of the proposed AFoFxNTSM is presented in this section. In particular, a concise discussion of the shortcomings of the suggested controller in terms of its parameters and stability analyses is included. In addition to this, potential applications of the proposed method to non-linear systems are also covered.

A comparison is made between the control strategy that has been suggested (AFoFxNTSM) and ATDENTSM, and the parameters of both systems are set in an appropriate way. Therefore, it is clear from looking at Figure 7 and Figure 8 that the suggested controller has the least amount of tracking errors and, accordingly, the least amount of time needed to converge. In addition, the control inputs of the joints can be noticed in Figure 9, and one can see the suggested method that was provided offers the control input that is the most smooth and efficient. Figure 11 and Figure 12 present the adaptive estimation, which demonstrates that there is no drifting problem with the adaptive rules. In addition, the root-mean-square (RMS) errors of the proposed AFoFxNTSM scheme are calculated as $\varepsilon_{1RMS}=0.0124$, $\varepsilon_{2RMS}=0.0125$, and $\varepsilon_{3RMS}=0.0123$, and the RMS errors of the ATDENTSM method are obtained as $e_{1RMS}=0.0317$, $e_{2RMS}=0.0189$, and $e_{3RMS}=0.0294$. Hence, both the simulation and the quantitative analyses demonstrate that the proposed method has a superior performance.

The parameters of the suggested control technique are chosen in accordance with the range that was provided, such as $\delta_{1}>0$, $\delta_{2}>0$, $\delta_{3}>0$, $0<\beta_{1}<1$, $\beta_{2}>1$, 0<α<1, $\delta_{4}>0$, $\delta_{5}>0$, $\delta_{6}>0$, $0<\varsigma_{1}<1$, $\varsigma_{2}>1$, and $0\leq \alpha_{1}<1$. In the event that these concerns are not considered, the closed-loop system does not continue to exhibit fixed-time stability. It is clear, based on the results of (31) and (46), that $T_{s1}$ and $T_{s2}$ are inversely proportional to $\delta_{i}$, whereas $\delta_{i}$ is proportional to τ(t) in (19) and (32). Therefore, in order to attain fixed-time convergence and closed-loop system stability at the same time, the suitable values of $\delta_{i}$ need to be set. These values determine the stability of the system. In addition, the ranges of the other parameters are known, which makes it possible to select the suitable value in a manner that is adequate. In fact, the scope of this work could be broadened to include the consideration of non-linearities that are not smooth for the non-linear systems.

## 7. Conclusions

An AFoFxNTSM was proposed in order to facilitate robotic manipulator trajectory tracking in the presence of uncertainties and external disturbances. An adaptive method was used in the construction of the proposed scheme so that it could estimate the unknown bounds of uncertainties and disturbances. This method also made it possible for the FoFxNTSM to achieve fixed-time convergence and tracking performance. On the 3−DOF PUMA 560 robotic manipulator, the AFoFxNTSM is implemented with known and unknown dynamics to demonstrate and explain the usefulness of the suggested technique. The findings of the simulation reveal that the suggested AFoFxNTSM method, compared with ATDENTSM, is superior in terms of response time and trajectory tracking errors, and has a higher capability to reject uncertainties and disturbances.

## Data Availability

Not applicable.
